# Biomimetic Recognition of SARS‐CoV‐2 Receptor‐Binding Domain N‐Glycans by an Antiviral Synthetic Receptor

**DOI:** 10.1002/cbic.202500106

**Published:** 2025-03-04

**Authors:** Carlo Santambrogio, Mirco Toccafondi, Lorena Donnici, Elisa Pesce, Raffaele De Francesco, Renata Grifantini, Erika Ponzini, Francesco Milanesi, Marco Fragai, Cristina Nativi, Stefano Roelens, Rita Grandori, Oscar Francesconi

**Affiliations:** ^1^ Dipartimento di Biotecnologie e Bioscienze Università di Milano-Bicocca Milan 20126 Italy; ^2^ Istituto Nazionale di Genetica Molecolare (INGM) ‘Romeo ed Enrica Invernizzi' Milan 20122 Italy; ^3^ Dipartimento di Scienze Cliniche e di Comunità Università degli Studi di Milano, Dipartimento di Eccellenza 2023-2027 Milan Italy; ^4^ Dipartimento di Scienze Farmacologiche e Biomolecolari Università degli Studi di Milano Milan Italy; ^5^ Dipartimento di Scienza dei Materiali Università di Milano-Bicocca 20125 Milan Italy; ^6^ Optics and Optometry Research Center (COMiB) Università di Milano-Bicocca 20125 Milan Italy; ^7^ Dipartimento di Chimica “Ugo Schiff” DICUS Università degli Studi di Firenze Firenze Italy; ^8^ Centro di Risonanze Magnetiche (CERM) Università degli Studi di Firenze Firenze Italy; ^9^ Consorzio Interuniversitario Nazionale per la Scienza e Tecnologia dei Materiali (INSTM) Firenze Italy; ^10^ Institute for Advanced Simulations Forschungszentrum Juelich 52428 Juelich Germany

**Keywords:** enveloped viruses, N-glycans, mannose recognition, synthetic receptors, native mass spectroscopy

## Abstract

Recognition of glycans by simple synthetic receptors is a key issue in supramolecular chemistry, endowed with relevant implications in glycobiology and medicine. In this context, glycoproteins featuring N‐glycans represent an important biological target, because they are often exploited by enveloped viruses in adhesion and infection processes. However, a direct evidence for their recognition by a synthetic receptor targeting N‐glycans is still missing in the literature. Using a combination of glycoengineering and mass spectrometry techniques, we present here the direct evidence of biomimetic recognition of complex‐type N‐glycans exposed on the receptor‐binding domain (RBD) of the wild‐type spike protein of SARS‐CoV‐2 by a biologically active, synthetic receptor.

## Graphical abstract

Glycan‐binding agents may be a key asset in the development of new antivirals against enveloped viruses. Using a combination of glycoengineering and mass spectrometry techniques, we present here direct evidence of biomimetic recognition of the N‐glycans exposed on the receptor‐binding domain (RBD) of the wild‐type spike protein of SARS‐CoV‐2 by a synthetic receptor targeting mannose and acting as antiviral.

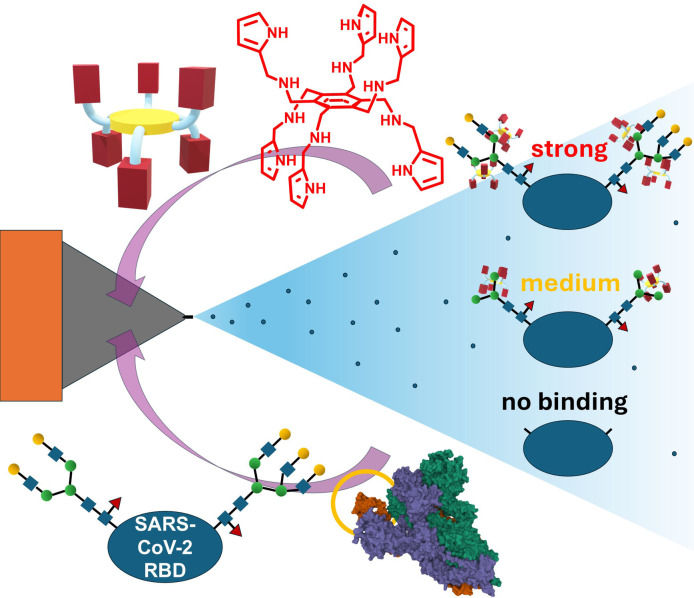

## Introduction

Carbohydrates assembled in glycans as glycoconjugates with lipids and proteins act as mediators in a variety of key biological processes, spanning from immune control and inflammation, to cell‐cell recognition and adhesion.^[1]^ Beyond physiological roles, biomolecular recognition of glycans also triggers several pathological pathways, such as those associated with cancer development and metastasis,[^2^, ^3^] or bacteria‐ and virus‐to‐host interactions, which lead to adhesion and infection by pathogens.^[4]^

Enveloped viruses, including those that have caused recent global health emergencies, such as HIV,[^5^, ^6^] Zika virus,^[7]^ Ebola virus[^8^, ^9^] and SARS‐CoV‐2,[^10^, ^11^] expose densely glycosylated surface glycoproteins, also known as spike proteins, which bind to specific target receptors on the surface of the host cell, enabling viruses to enter and replicate.^[12]^ Glycosylation of surface proteins provides a double advantage to viruses, as it facilitates binding to the host target receptor, and masks the underlying immunogenic amino acids, evading the host immune surveillance.^[13]^

N‐linked glycans, carrying a mannose core (Man_3_GlcNAc_2_ or Man_3_GlcNAc_2_Fuc), are central to the infection mechanism of various enveloped viruses and have been proposed as targets for new therapeutic strategies. For instance, oligomannose N‐glycans on gp120 of HIV facilitate the viral entry by binding to the receptor domain of the DC‐SIGN protein on the host cell,^[9]^ while oligomannosides on the spike protein of SARS‐CoV‐2 are known to stabilize the protein in the “up” conformation, which is required for binding to the host cell angiotensin converting enzyme 2 (ACE2).[^14^, ^15^]

Because of the high mutation frequency of SARS‐CoV‐2, glycan‐binding agents may provide a key asset for the development of new strategies to reduce the infection burden and disease severity of COVID‐19 and, more generally, may represent versatile antivirals that disrupt infection pathways relying on glycans recognition.^[16]^ In this respect, it is not surprising that lectins and therapeutic antibodies, interacting with the oligomannosides of the HIV gp120 protein, have elicited a broad‐spectrum neutralization against HIV.[^17^, ^18^] Although lectins and antibodies targeting carbohydrates recognize specific glycans, lectin poor specificity and the intrinsic immunogenicity, as well as antibodies complex manufacturing process, make their use in therapy and diagnostic extremely challenging.[^19^, ^20^]

To tackle this problem, artificial carbohydrate binding agents (CBAs) have been developed, including molecular‐imprinted polymers, aptamers and synthetic receptors. Relying exclusively on the same non‐covalent interactions used by lectins and antibodies, synthetic receptors are the most studied class of biomimetic CBAs.[^21^, ^22^] Although only few of them have been tested as potential biologics, promising results were obtained against enveloped viruses, including HIV and Zika.[^21^, ^23^] In this context, some authors have developed a family of aminopyrrolic receptors recognizing mannosides by means of a combination of hydrogen bonds and CH‐π interactions.[^24^, ^25^, ^26^, ^27^, ^28^, ^29^] This class of artificial mannose‐binding agents has proved to possess biological activity in a variety of contexts involving high‐mannose‐type glycans.[^30^, ^31^, ^32^] When tested for antiviral activity, aminopyrrolic receptors showed to effectively inhibit the HIV infection by targeting the highly mannosylated glycoprotein gp120 of the viral envelope.[^23^, ^33^]

Prompted by the outbreak of the COVID‐19 pandemic, a selection of the most effective aminopyrrolic receptors was investigated against SARS‐CoV‐2.^[34]^ A lead compound devoid of toxicity (IDS060) emerged from the study, capable of inhibiting the SARS‐CoV‐2 infection by binding to the spike protein, thus preventing the viral entry. IDS060 showed a broad‐spectrum neutralizing activity toward several variants of the spike protein.

IDS060 was found to bind to the receptor‐binding domain (RBD) of the wild‐type spike protein, which shows a prevalence of complex‐type N‐glycans, inhibiting binding of the virus to the human ACE2 receptor. However, inhibition activity was also observed for variants of the spike protein for which little or no binding of IDS060 to RBD was observed, suggesting the occurrence of multiple interaction sites; therefore, inhibition of the infection through multiple mechanisms may be inferred. Due to the heavy glycosylation of the spike protein and the presence of N‐glycans featuring a mannosilated core, it was proposed that the activity of IDS060 is ascribed to its ability to recognise these mannosilated glycans. Although the data collected supported this hypothesis,^[34]^ direct evidence of molecular recognition of the N‐glycans of the spike protein by IDS060 has not yet been found.

Mass spectrometry (MS) is a powerful technique to study the interaction of small molecules with complex systems, such as glycoproteins, and is playing an increasingly important role in biochemistry and structural biology.^[35]^ A prominent advantage is direct detection of non‐covalent complexes, dissecting mixtures in all their components in terms of molecular mass, stoichiometry and conformation. The so‐called “native MS” approach analyzes protein conformations and supramolecular complexes by preserving non‐covalent interactions in the gas phase, thus enabling structural and functional investigation.^[36]^ MS analysis of highly glycosylated proteins is challenged by mass heterogeneity and poor desolvation/ionization efficiency.^[37]^ These drawbacks impact on native‐MS measurements, strongly affecting resolution and sensitivity in detecting intact glycosylated proteins and protein complexes.^[38]^ Several strategies have been described to either bypass or overcome these difficulties. One approach is glycoengineering, either by inhibiting the post‐translational modification at specific stages *in vivo*, or by enzymatic trimming of glycan antennas *in‐vitro*,^[39]^ resulting in more homogeneous samples for MS analysis. Another approach consists in enhancing performance of MS analysis of heterogeneous samples by the “limited charge reduction” method.^[40]^ In this case, electron‐capture, or electron‐transfer dissociation (ECD or ETD) techniques, are used in a top‐down MS/MS scheme, in which the product ions are still intact proteins with reduced charge states and reduced mass noise. Both these approaches have been applied successfully to native MS analysis of the SARS‐Cov‐2 spike protein, describing protein‐protein and protein‐drug interactions.[^39^, ^41^, ^42^] In this regard, a very recent paper describes the characterization of the glycoform distribution of the SARS‐CoV‐2 spike protein using native mass spectrometry combined with charge reduction methods.^[43]^

In this paper, a combination of glycoengineering and MS techniques has been used to characterize the binding interaction between IDS060 and the glycosylated RBD of the SARS‐CoV‐2 wild‐type spike protein. The results reported herein give the unprecedented proof of the recognition of a glycoprotein by a biologically active synthetic receptor targeting the N‐glycans, opening the way for further development of a promising class of broad‐spectrum antiviral agents.

## Results and Discussion

**Scheme 1:**
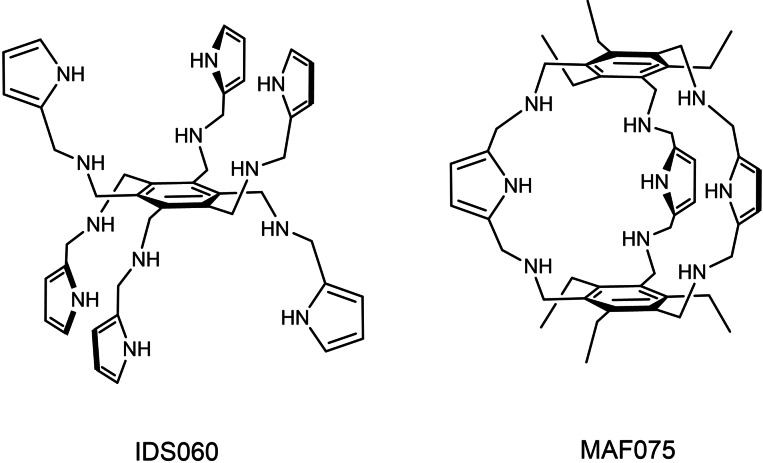
Chemical structure of IDS060 and MAF075.

**Figure 1:**
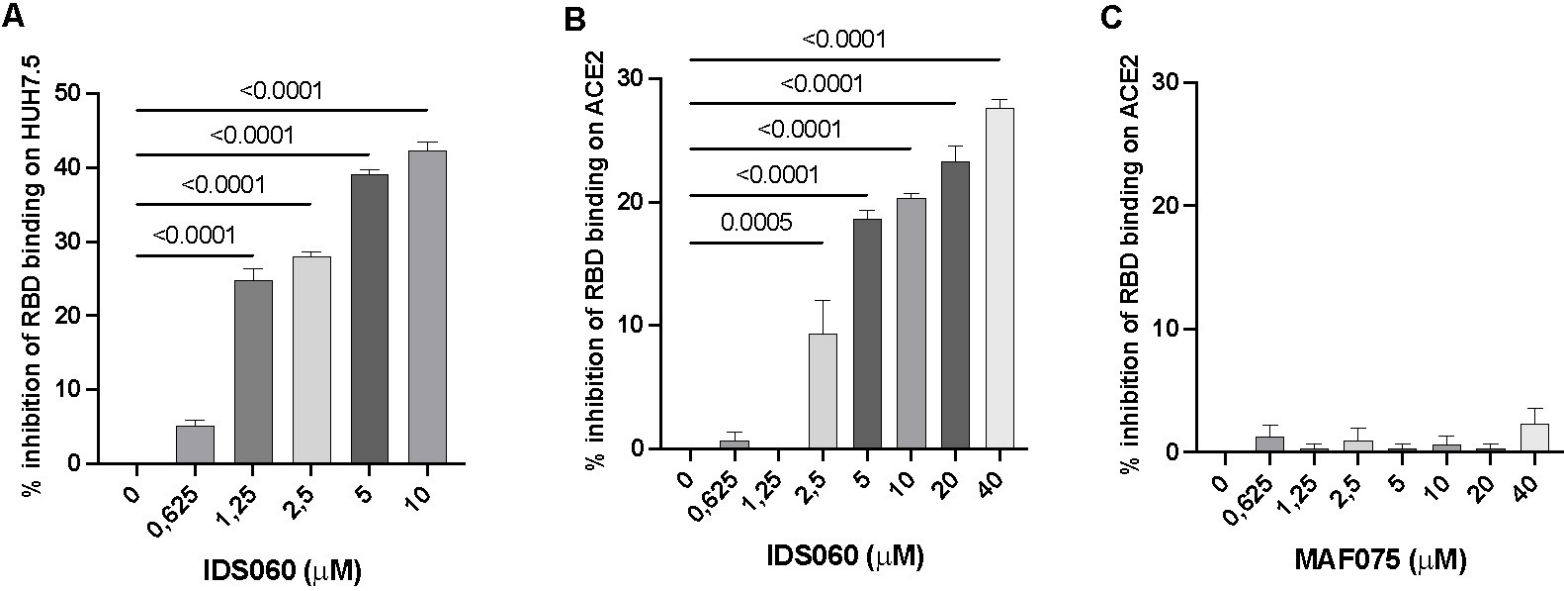
IDS060 specifically recognises RBD and inhibits binding to ACE2. A) Competitive flow cytometry. The HUH7.5 cell line, expressing the ACE2 receptor, was incubated with the biotinylated recombinant RBD with increasing concentrations of IDS060. Streptavidin conjugated to PE was used for detection. B−C) Competitive ELISA assay. Plates coated with recombinant ACE2 were incubated with biotinylated recombinant RBD with increasing concentration of IDS060 (B) or MAF075 (C), using HRP‐streptavidin for binding detection. In A, B and C, the IDS060 ability to inhibit RBD‐ACE2 binding was measured as compared to samples incubated without IDS060 and expressed as average percent value of three replicates±SD. The statistical test that was applied for all analyses is Ordinary one‐way ANOVA and was calculated with Prism 9. Each sample was analyzed in triplicate (n=3). Error bars represent the standard deviation (SD) of the mean. Figure 1A has been repeated based on previously published work.^[34]^

We first investigated whether IDS060 (Scheme 1), prepared according to a previously reported procedure,^[30]^ is able to inhibit the binding of RBD to the ACE2 receptor in a way specifically related to its structural features. Therefore, we performed a

competitive binding assay by flow cytometry on the ACE‐2‐expressing HuH7.5 cells with biotinylated recombinant RBD, in the presence of increasing concentration of IDS060 (0.625‐10 μM) and measured the inhibition of surface binding of RBD on the cells, with respect to untreated sample devoid of IDS060. Correspondingly, we tested the artificial receptor MAF075 (Scheme 1) specific for glucose, which is not present in the structure of N‐glycans. MAF075, prepared according to a previously reported procedure,^[44]^ is structurally related to IDS060 because of the aminopyrrolic hydrogen‐bonding groups and the benzene scaffold.[^44^, ^45^, ^46^] We found that IDS060 inhibits binding of RBD to the receptor (inhibition varying from 40 % to 25 %) (Figure 1A), whereas the activity of MAF075 could not be assessed due to cell toxicity, even at the lowest tested concentration (at 1 μM cell viability was 10 %). To address this issue, we tested the activity of IDS060 and MAF075 by competitive ELISA extending the concentration range up to 40 μM, using recombinant RBD and ACE2 proteins. IDS060 was confirmed to inhibit binding between ACE2 and RBD (Figure 1B), whereas MAF075 showed no inhibitory activity, even at 40 μM, therefore excluding any possible interaction with RBD (Figure 1C).

**Figure 2:**
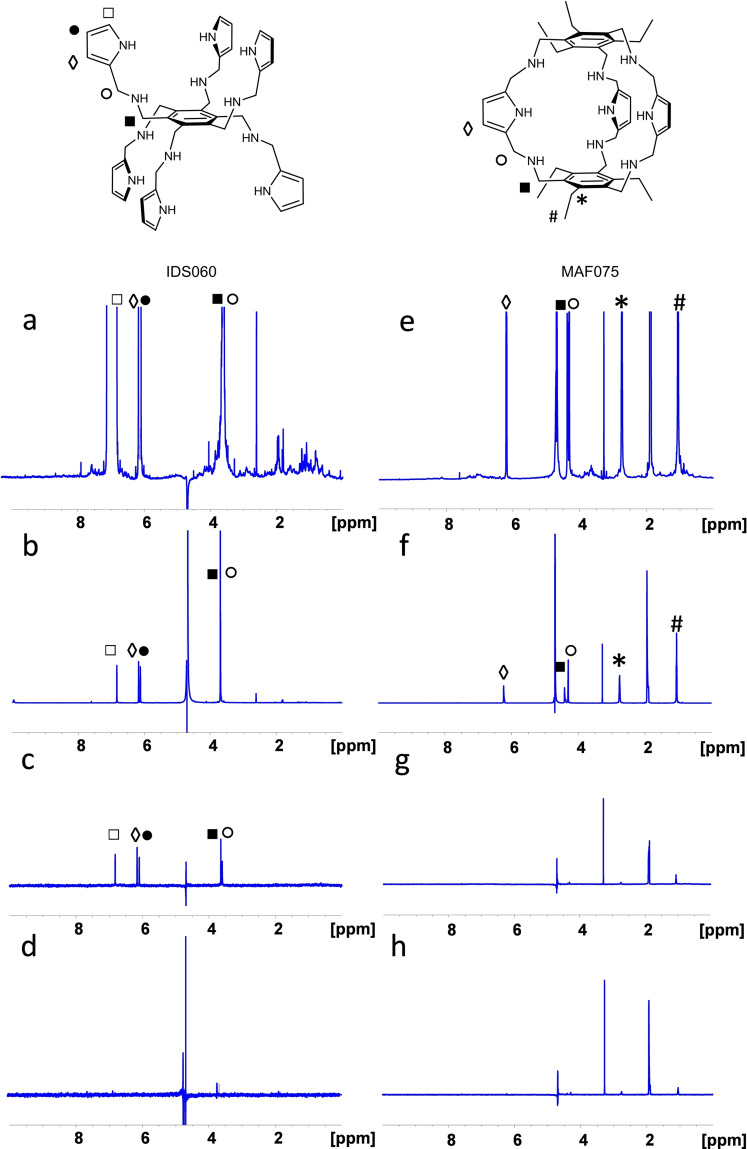
Binding analysis of IDS060 and MAF075 to RBD by saturation transfer difference (STD) experiments. ^1^H NMR spectra of IDS060 and MAF075 (both 800 μM) in the presence (A, C and E, G respectively) and in the absence (B, D and F, H respectively) of RBD (20 μM). (A and E) Reference spectra of IDS060 and MAF075+RBD saturated off‐resonance at δ=−40 ppm. The spectra, showing signals from both partners, were scaled up to the protein intensity; (B and F) Reference spectra of IDS060 and MAF075 saturated off‐resonance at δ=−40 ppm; (C and G) Difference spectra (STD) between IDS060 and MAF075 + RBD saturated off‐resonance at δ=−40 ppm, and on‐resonance at δ=−2 ppm; (D and H) difference spectra (STD) between IDS060 and MAF075 saturated off‐resonance at δ=−40 ppm, and on‐resonance at δ=−2 ppm. Signals assignment of IDS060 and MAF075 are indicated in the spectra. Residual signals of MAF075 are depleted, both, in the presence or in the absence of the protein (difference spectra G and H are comparable), showing no evidence of binding.

The lack of interaction of MAF075 with RBD was further confirmed by saturation transfer difference (STD) NMR experiments, under the same conditions used for IDS060‐RBD binding analysis in a previous work.^[34]^ Indeed, two identical STD spectra were obtained in the presence and in the absence of the protein, in which only residual solvent peaks are evident (Figure 2, in comparison with IDS060‐RBD STD spectra from ref. [34]).

MS‐based methods were then applied to seek direct evidence for complex formation between IDS060 and RBD and to characterize and compare RBD preparations presenting different extents of glycosylation. The fully N‐glycosylated protein was expressed in the Expi293F cell line, whereas a form with only a partial‐N‐glycosylation, was expressed in the Expi293F GnTI cell line, lacking N‐acetylglucosaminyl‐transferase I (GnTI) enzyme and limiting N‐glycosylation to the Man_3_GlcNAc_2_ stage. Finally, a N‐deglycosylated form of the protein was obtained from fully N‐glycosylated RBD treated with PNGaseF, resulting in a complete removal of N‐glycosylations.

**Figure 3:**
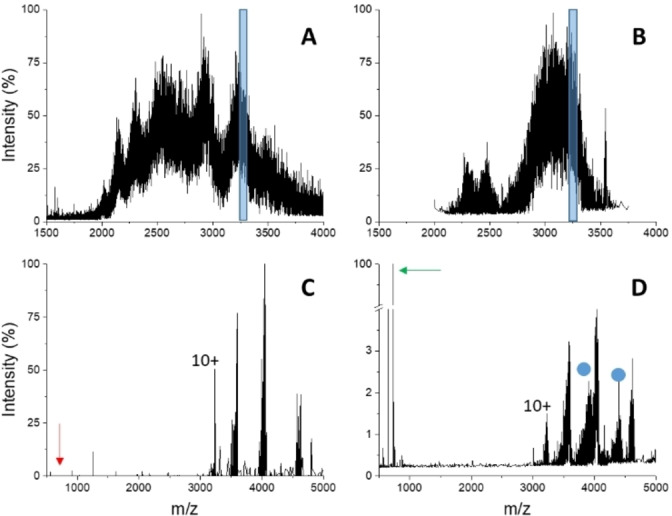
Nano‐ESI‐MS spectra (A, B) and limited charge reduction by ETD (C, D) of 15 μM fully N‐glycosylated RBD in 50 mM ammonium bicarbonate pH 7.9. A, C) 0 μM IDS060; B, D) 150 μM IDS060. The ion‐selection windows applied for limited charge reduction by ETD are shown as blue boxes in panels A and B (parent ion 10+). Arrows point to the signal of free, singly charged IDS060 (red: theoretical position; green: detected peak). Putative signals of the RBD‐IDS060 complex are labelled by blue circles.

The pure, recombinant protein has been analyzed by native MS under non‐denaturing conditions (Figure 3A). As expected, the spectrum shows poorly resolved peaks that do not allow unambiguous charge assignment, an effect that is likely due to the presence of complex‐type glycans. By comparing buffered solutions with denaturing conditions (Figure S2A), a shift of the peak envelopes towards higher charge states can be observed in the latter spectra, consistent with protein unfolding, along with a minor improvement in spectral quality. This result suggests that the protein analyzed by native MS maintains a compact conformation. The addition of a ten‐fold molar excess of IDS060 induced a further loss in spectrum quality and led to completely unresolved ion signals (Figure 3B). Nonetheless, this effect can be taken as a first indication of protein‐synthetic receptor interactions taking place under the employed experimental conditions.

In order to improve the quality of native‐MS spectra and better investigate the binding properties of IDS060, limited charge reduction was performed by ETD (Figure 3C, D) as described in Methods. The product ions gave well‐resolved peak envelopes that have been deconvoluted, yielding charge and mass values. The sample in the absence of the synthetic receptor from non‐denaturing conditions (Figure 3C) presents signals that can be ascribed to the protein as such, with a resulting mass of 32,211±36 Da. The same applies to the protein in the absence of the synthetic receptor from denaturing conditions (Figure S2D). This condition yielded cleaner spectra and a mass value of 31,974±25 Da. The protein average mass calculated from the amino acid sequence was 26,583.92 Da. These results indicate a mass shift of approximately 5390 Da due to glycosylation, consistent with the reported modifications of the SARS‐Cov‐2 RBD by N‐ and O‐glycosylation.[^47^, ^48^, ^49^] On the other hand, charge reduction by ETD of selected protein ions from the 1 : 10 protein/synthetic receptor mixture gave rise to signals in the protein region, plus an intense peak at *m/z* 727.48±0.05 (Figure 3D) corresponding to the 1+ state of the ligand (727.4673 Da, calculated mass of the monoisotopic, protonated form) dissociating from the protein upon the limited charge reduction procedure. This result provides direct evidence of RBD‐IDS060 complex formation. Due to the improved resolution achieved by limited charge reduction, peaks in the protein region reveal a double series, corresponding to 32,326±87 and 35,264±39 Da. This double peak envelope can be ascribed to the presence of the free protein (the former) and some residual complex upon ETD charge reduction (the latter). The mass shift was consistent with a 1 : 4 (±0.2) stoichiometry and indicates binding of fully N‐glycosylated RBD to the IDS060 ligand.

Similar results have been obtained using precursor ions from different charge states (data not shown). A rough quantitation of the ratio between the signal of the complex and the total protein intensity for the 9+ charge state in Figure 3D (peak with highest apparent binding) gave 36 % residual protein saturation among the ETD products. Considering that in‐source and ETD‐triggered dissociation phenomena of the complex are probable, such a value underestimates the extent of binding in the original sample and could be taken as an indicative lower limit.

**Figure 4:**
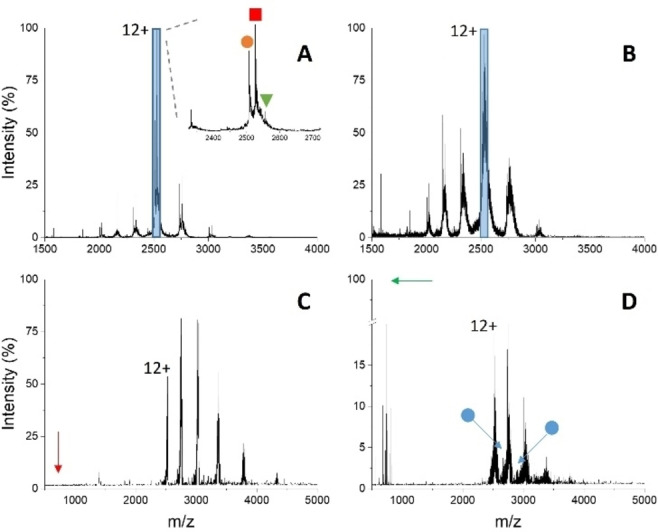
Nano‐ESI‐MS spectra (A, B) and limited charge reduction by ETD (C, D) of 15 μM partially‐N‐glycosylated RBD (expressed in GnTI cell line) in 50 mM ammonium bicarbonate pH 7.9. A, C) 0 μM IDS060; B, D) 150 μM IDS060. The ion‐selection windows applied for limited charge reduction by ETD are shown as blue boxes in panels A and B (parent ion 12+). The inset in panel A highlights protein mass heterogeneity: 30,076 Da (orange circle), 30,305 Da (red square) and 30,670 Da (green triangle). Arrows point to the signal of free, singly charged IDS060 (red: theoretical position; green: detected peak). In panel D, putative signals of the RBD‐IDS060 complex are labelled by blue circles.

The limited charge reduction method has been combined with a glycoengineering approach to obtain a more homogeneously glycosylated species for MS analysis. Thus, the RBD protein has been expressed in the GnTI cell line, resulting in a partially‐N‐glycosylated product.^[39]^ Consistently, the spectra show increased resolution compared to the fully N‐glycosylated protein (Figure 4). Again, shift and broadening of the CSD (main charge state from 12+ to 14+), comparing non‐denaturing and denaturing conditions, (Figure 4A, S2B) indicate that the protein maintains a compact conformation during native‐MS analysis. The increased spectral quality allows charge assignment without the ETD step. However, some mass heterogeneity persists, with main peaks corresponding to 30,076±1 Da, 30,305±1 Da and 30,670±1 Da, as calculated from denaturing conditions (Figure S2B). These values imply a residual contribution from glycan chains, ranging approximately from 3500 to 4100 Da, compatible with two Man_3_GlcNAc_2_Fuc modifications (911 Da each). The additional mass shift of approximately 1678–2278 Da, relative to the naked polypeptide chain, could be due to O‐glycosylations, which are not affected by the GnTI enzyme mutation. O‐glycans on residues T323 and S325 of native spike have been reported.^[47]^

The addition of IDS060 at 1 : 10 protein‐ligand ratio resulted in lower spectral quality but was still compatible with charge assignment (Figure 4B). Also in this case, limited charge reduction by ETD enabled analysis of protein‐ligand interaction. The molecular weights obtained under denaturing conditions with and without charge reduction (Figure S2B, S2E) are in close agreement (within 55 ppm discrepancy). The MS/MS spectra obtained from the samples of RBD alone do not show any evidence of signals corresponding to the ligand in either native or denaturing conditions (Figure 4C, S2E). Instead, results from the 12+ peak of the RBD : IDS060 1 : 10 mixture under non‐denaturing conditions show the signal of the released ligand, in addition to peaks in the protein region (Figure 4D). Similar to the results shown in Figure 3, the double series of protein peaks likely represents the free and bound species, although with lower relative intensity of the residual complex upon ETD compared to the fully N‐glycosylated protein. The corresponding mass values of 30,197±123 and 32,015±112 Da imply a mass shift of 1818 Da. This result indicates that the synthetic receptor binds to the core structure of the RBD N‐glycans as well, although with a lower stoichiometry of approximately 1 : 2 (1 : 2.5±0.5) instead of 1 : 4. The extent of protein saturation, calculated from the 12+ signal of the MS/MS spectrum, is also lower than that of the native protein, whereby the value calculated from the spectrum of Figure 4D is 19 %.

**Figure 5:**
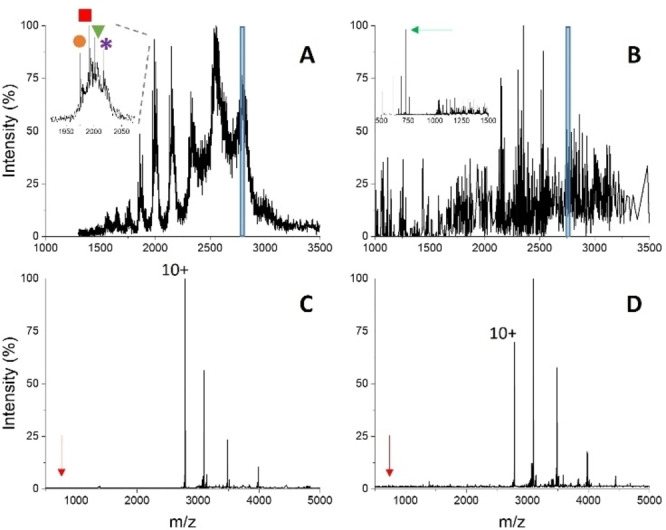
Nano‐ESI‐MS spectra (A, B) and limited charge reduction by ETD (C, D) of 15 μM N‐deglycosylated RBD (PNGaseF‐treated) in 50 mM ammonium bicarbonate pH 7.9. A, C) 0 μM IDS060; B, D) 150 μM IDS060. The ion‐selection windows applied for limited charge reduction by ETD are shown as blue boxes in panels A and B (parent ion 10+). The inset in panel A highlights protein mass heterogeneity: 27,645 Da (orange circle), 27,874 Da (red square), 28,008 Da (green triangle) and 28,237 Da (purple asterisk). The inset in panel B highlights the presence of free IDS060. Arrows point to the signal of free, singly charged IDS060 (red: theoretical position; green: detected peak).

To further assess the role of the glycan chains in IDS060 binding, the RBD protein has been N‐deglycosylated by PNGaseF treatment. The spectra of the enzymatically treated protein under native and denaturing conditions are reported in Figure 5A and S2 C, respectively. The spectra display improved quality, compared to Figure 3, consistent with removal of the N‐linked glycans. As highlighted in the inset of Figure 5A, the sample exhibits some mass heterogeneity, with main masses of 27,645±3, 27,874±3, 28,008±3 and 28,237±3 Da. These values represent shifts of 1,061, 1,290, 1,424, 1,653 Da relative to the molecular weight calculated from the amino acid sequence. Such mass shifts could represent the contribution of the two O‐glycan chains in RBD, which are not affected by the PNGaseF treatment.

To support the interpretation of the above results, the PNGaseF‐treated sample has also been investigated by a bottom‐up approach. Tryptic peptides were prepared by standard procedures, processed by an LC–MS/MS workflow, and identified by a software dedicated to glycopeptide analysis, searching for 32 unique masses of human O‐glycans. The results are reported in Table S1. Glycosylated peptides were identified with high confidence, spanning the known O‐glycosylation sites T323 and S325. The modifications could be detected in most cases by direct spectral evidence at the MS/MS level and, in a few instances, by inference from the precursor‐ion mass shift. These results indicate that the main four signals in the native‐MS spectrum of Figure 5A can be interpreted by the mass heterogeneity of the O‐glycan chains.

Ligand binding was tested by native MS as above, with 1 : 10 protein‐IDS060 mole ratio. Addition of ligand resulted in poorer spectral quality (Figure 5B). However, it can be appreciated that no new peak envelope ascribable to the protein‐ligand complex appears upon charge reduction by ETD (Figure 5D), indicating that binding does not occur with the PNGaseF‐treated protein. The combined top‐down and bottom‐up MS analyses of the N‐deglycosylated protein reported here indicate that the RBD O‐glycans do not promote interaction with IDS060.

**Figure 6:**
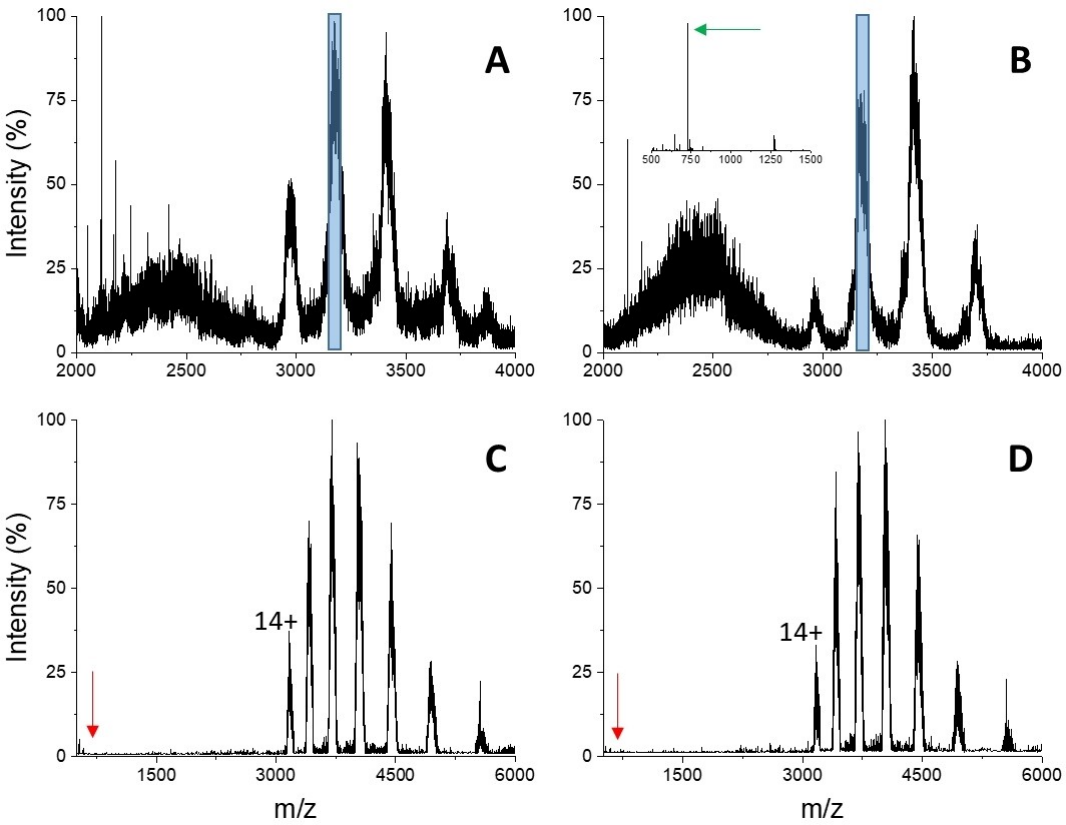
Nano‐ESI‐MS spectra (A, B) and limited charge reduction by ETD (C, D) of 15 μM ovalbumin in 50 mM ammonium bicarbonate pH 7.9. A, C) 0 μM IDS060; B, D) 150 μM IDS060. The ion‐selection windows applied for limited charge reduction by ETD are shown as blue boxes in panels A and B (parent ion 14+). The inset in panel B highlights the presence of free IDS060. Arrows point to the signal of free, singly charged IDS060 (red: theoretical position; green: detected peak).

To test the specificity of glycoprotein recognition by IDS060, ovalbumin was employed as an alternative N‐glycosylated protein. The results are reported in Figure 6. The poor resolution of the MS spectra is consistent with the glycosylated nature of the protein (Figure 6A). The addition of 1 : 10 molar excess of IDS060 did not elicit significant spectral changes (Figure 6B). Limited charge reduction by ETD resulted in the expected resolution enhancement, yielding deconvoluted, average mass values of 44,379±40 Da from non‐denaturing conditions (Figure 6C) and 44,166±15 Da from denaturing conditions (Figure S3). The average mass calculated by sequence is 42,881 Da, indicating a glycan moiety of about 1285 Da in the protein preparation employed here, compatible with a single N‐glycosylation.^[50]^ Upon the addition of the ligand, the MS/MS spectra appeared unchanged, whereby signals ascribable to the free ligand, or to the putative protein‐ligand complex, could not be identified (Figure 6D). Similar results were observed by selecting precursor ions from the 13+, instead of 14+, charge state (data not shown). These results indicate that IDS060 does not bind to glycosylated ovalbumin, suggesting that the specificity of IDS060 for the N‐glycan chains of RBD may result from a combination of factors including the structure of the recognised glycans, the accessibility of the glycans on the glycoprotein surface and on the possible presence of nearby amino acids that hinder or stabilise one complex with respect to the other.

## Conclusions

In conclusion, the reported evidence indicates that IDS060 selectively binds to the N‐glycan chains of RBD and that the core structure of the glycan is the minimal requirement for recognition, while full‐length antennas are important for higher intensity of the complex peak observed. The observed 1 : 4 stoichiometry suggests that 2 ligand molecules are bound to each glycan antenna, whereas the lower 1 : 2 stoichiometry observed with RBD expressed in GnTI cell lines suggests that recognition most likely involves multiple interactions extending beyond the Man_3_GlcNAc_2_ core to the neighbouring carbohydrates. These results not only provide insight into the mechanism of action of IDS060 as an antiviral but also represent an unprecedented observation of biomimetic recognition of a glycoprotein by a synthetic receptor, opening the way to further development of synthetic receptors for carbohydrates to target N‐glycans for biofunctionality.

## Supporting Information

The authors have cited additional references within the Supporting Information.^[51]^ Methods, Figures S1–3, and Table S1 are included.

## Conflict of Interests

The authors declare no conflict of interest.

1

## Supporting information

As a service to our authors and readers, this journal provides supporting information supplied by the authors. Such materials are peer reviewed and may be re‐organized for online delivery, but are not copy‐edited or typeset. Technical support issues arising from supporting information (other than missing files) should be addressed to the authors.

Supporting Information

## Data Availability

The data that support the findings of this study are available in the supplementary material of this article.
