# Supplementary material for: Biomimetic Recognition of SARS‐CoV‐2 Receptor‐Binding Domain N‐Glycans by an Antiviral Synthetic Receptor
Source: Chembiochem. 2025 Mar 4;26(7):e202500106. doi: 10.1002/cbic.202500106 (PMC12002116; doi:10.1002/cbic.202500106)
Supplement: Supplementary file 1 — Supporting Information [file CBIC-26-e202500106-s001.pdf]

ChemBioChem

Supporting Information

Biomimetic Recognition of SARS-CoV-2 Receptor-Binding Domain N-Glycans by an Antiviral Synthetic Receptor

Carlo Santambrogio, Mirco Toccafondi, Lorena Donnici, Elisa Pesce, Raffaele De Francesco, Renata Grifantini, Erika Ponzini, Francesco Milanesi, Marco Fragai, Cristina Nativi, Stefano Roelens, Rita Grandori,* and Oscar Francesconi*

Supporting Information

Methods

No unexpected or unusually high safety hazards were encountered.

Expression and purification of RBD as fully N-glycosylated, with partial-N-glycosylations or N-deglycosylated

The sequence of the RBD domain of the wild-type SARS-CoV-2 Spike protein (residues 319-541 of Spike protein, UniProt P0DTC2) with a C-terminal His-tag was synthesized, codon-optimized, cloned into the pcDNA-3.4 vector and sequenced at GenScript (Netherland). Fully N-glycosylated RBD was expressed by transfection of the Expi293F cell line (Thermo Fisher Scientific). Transfection of the Expi293F GnTI cells (Thermo Fisher Scientific), lacking the N-acetylglucosaminyl-transferase I enzyme, ensured the production of partially-N-glycosylated RBD.

In brief, both cell lines were cultured at 37 °C and 8% CO_2 for transient expression for five days. Clarified culture supernatant was loaded on Nickel affinity resin (HisTRAP FF, Cytiva) equilibrated with 20 mM Tris-HCl pH 8.0, 150 mM NaCl and eluted in 20 mM Tris-HCl pH 8.0, 150 mM NaCl, 300 mM imidazole. Eluted proteins were concentrated with a 10 kDa MWCO centrifugal filter (Amicon Ultra, Millipore Sigma), and purified by size-exclusion chromatography (Superdex 200-Increase 10/300 GL, Cytiva) in PBS or 50 mM NH_4HCO_3 pH 7.9.

In addition, purified, fully N-glycosylated RBD was treated by N-glycosidase F (PNGaseF, NEBiolab) for a complete enzymatic removal of N-linked oligosaccharides. PNGaseF treatement was performed following manufacturer instruction in PBS at 30 °C over-night. N-deglycosylated RBD was concentrated with a 10 kDa MWCO centrifugal filter and loaded on Superdex 200-Increase 10/300 GL column in PBS or 50 mM NH_4HCO_3 pH 7.9.

Fully N-glycosylated, partially-N-glycosylated and N-deglycosylated proteins were loaded on an SDS-PAGE under reducing conditions to check their apparent molecular weight and purity (Figure S1).

Competitive flow cytometry binding assay.

Huh7.5 (RRID:CVCL_7927) cell line was a gift of Prof. Charles M. Rice, Laboratory of Virology and Infectious Disease, The Rockefeller University, NY, USA. The HUH7.5 cell line expressing the ACE2 receptor was seeded in 96-well plates (10000 cells/well), and incubated with the biotinylated recombinant RBD (6.25 μg/mL) in the presence of increasing concentrations of IDS060 or MAF075 (0 - 10 μM) in PBS for 1 hour at 37 °C. The unbound protein was removed by washing twice with PBS. Cells were then incubated with streptavidin conjugated to phycoerythrin (PE) and analysed by flow cytometry on a FACSCanto-II (BD Biosciences). Data were analyzed using FlowJo software version 10 (BD Biosciences). The binding inhibition was normalized referring to HuH7.5 cells incubated only with RBD, which was considered as 100% binding.

Competitive ELISA assay

96-well microwell plates were coated with 50 ng/well recombinant ACE2 in PBS 1x and incubated overnight at 4 °C. After blocking, the plates were washed with 0,05% PBS-T and biotinylated recombinant RBD (6.25 μg/mL) was added to each well with increasing concentrations of IDS060 or MAF075 (0 - 40 μM diluted in PBS starting from stock solutions in DMSO) for 1 h at 37 °C. HRP-

streptavidin was used to detect the binding of RBD to ACE2. The binding inhibition was normalized referring to ACE2 samples incubated with only RBD, considered as 100% binding.

NMR spectroscopy

The saturation transfer difference (STD) spectra were recorded at 298 K in PBS with 10% D_2O on a Bruker Avance NEO NMR spectrometer operating at 950 MHz 1H Larmor frequency, equipped with a cryogenically cooled probe. All spectra were processed with the Bruker TOPSPIN software packages. The saturation transfer difference (STD) experiments were acquired with a pseudo-2D pulse sequence and 512 scans on a solution containing recombinant RBD to ID060 or MAF075 molar ratio of 1:40. Saturation was achieved with a cascade of Gaussian pulses of 50 ms duration that were applied for 2.5 s. Selective saturation of the Spike RBD was achieved by applying the on-resonance irradiation at -2 ppm, near to protein signals but far from those of the ligands IDS060 and MAF075. The off-resonance irradiations were applied at -40 ppm.

Mass spectrometry

Native MS experiments were performed on an Orbitrap Fusion mass spectrometer equipped with a static nano-electrospray ion source (Thermo Fisher Scientific,Waltham, MA, USA), employing metal-coated borosilicate capillaries with a medium-length emitter tip of 1 μm internal diameter (Thermo Fisher Scientific, Waltham, MA, USA). The main instrumental parameters were set as follows: resolving power at m/z 200, R=120,000; IRM pressure, 3 mTorr (intact protein mode); ion spray voltage, 1.1–1.2 kV; ion-transfer tube temperature, 275 °C; in-source fragmentation, 0-50 V; AGC target, 4×10^5; maximum injection time, 100 ms. Limited charge reduction analysis at selected m/z values was performed by ETD, employing a reaction time of 10-20 ms and an isolation width of 5-20 m/z. Final spectra were obtained by averaging the signal over 30 s acquisition time. The peptides for the bottom-up MS analysis were generated by trypsin digestion (16h incubation at 37 °C), desalted by Ziptip columns (Millipore, Burlington, MA, USA) and measured by reversed phase LC-MS/MS on the same Orbitrap instrument hyphenated with a EASY-nLC1000 unit. A 50-cm EASY-Spray C18 column (Thermo Fisher Scientific, Waltham, MA, USA) was eluted by a 60 minutes 5-75% acetonitrile gradient.

Statistical Analysis

Native MS spectra were obtained by averaging the signal (ion counts) over 30 s acquisition time, and normalizing peak intensities relative to the most intense signal. The molecular mass of detected species is calculated as the weighted average over the detected charge-state distribution. Quantitative data are expressed as the mean ± standard deviation over at least two measurements (two independent batches), each run in technical duplicate. The peptides identification and the determination of their glycosylation state in bottom-up MS experiments was given by the program O-Pair[51]. The confidence level in Table S1 is defined as follows: 1, all glycans are localized with direct spectral evidence; 2, only one glycan is localized with direct spectral evidence (and the other one inferred by exclusion); 3, no glycans are localized.

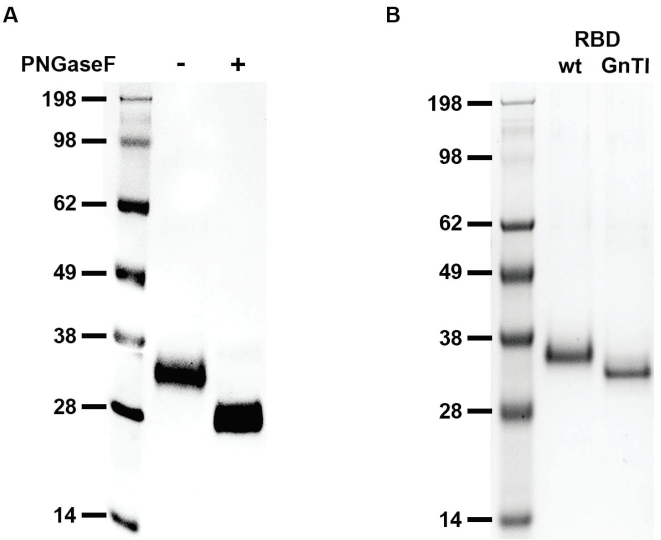

Figure S1. SDS-PAGE of fully N-glycosylated, partially-N-glycosylated and N-deglycosylated RBD. A) Fully N-glycosylated RBD before and after PNGaseF treatment. The untreated protein shows an apparent molecular weight higher than the theoretical one due to the glycosylations. The band of the N-deglycosylated protein, instead, is close to the expected molecular weight of 26.6 kDa. B) RBD expressed in Expi293F and Expi293F GnTI cell lines. The partially-N-glycosylated protein shows a band with an intermediate apparent molecular weight, between the fully N-glycosylated and the N-deglycosylated protein.

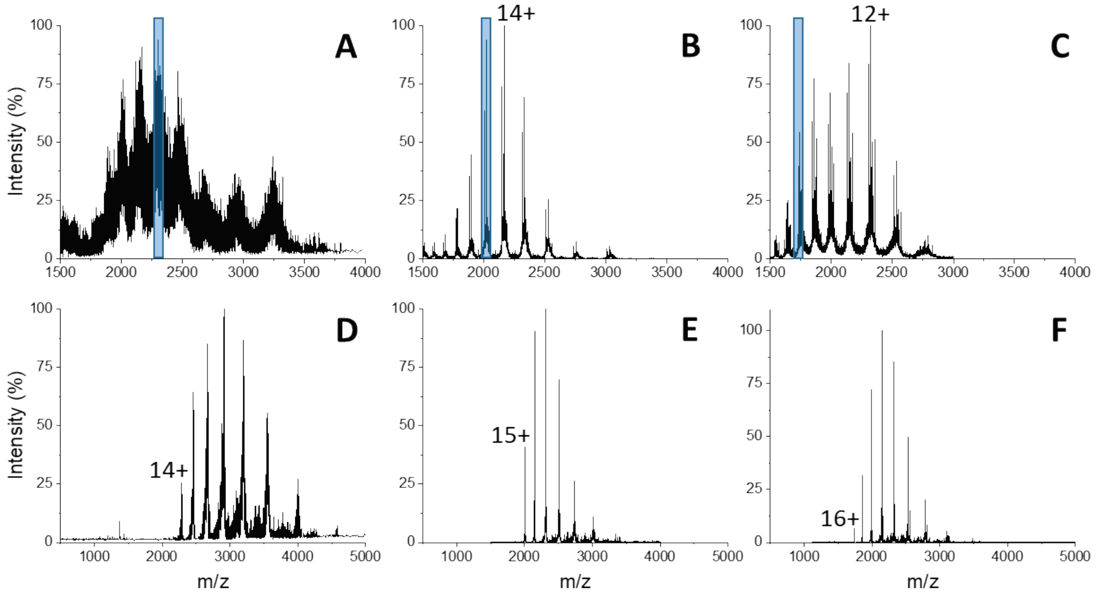

Figure S2. Nano-ESI-MS spectra (A-C) and limited charge reduction by ETD (D-F) of 15 µM protein in 50% acetonitrile 0.5% formic acid. A, D) Fully N-glycosylated RBD; B, E) partially-N-glycosylated RBD; C, F) N-deglycosylated RBD. The ion-selection windows applied for limited charge reduction by ETD are shown as blue boxes in panels A-C.

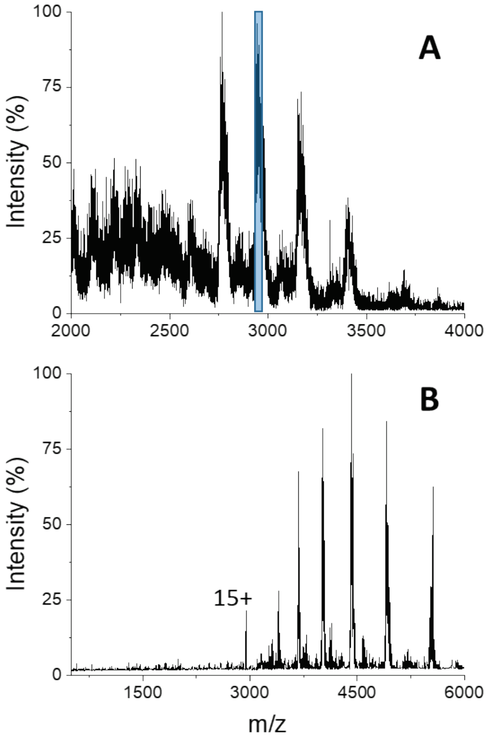

Figure S3. Nano-ESI-MS spectrum (A) and limited charge reduction by ETD (B) of 15 µM ovalbumin in 50% acetonitrile 0.5% formic acid. The ion-selection window applied for limited charge reduction by ETD is shown as blue box in panel A.

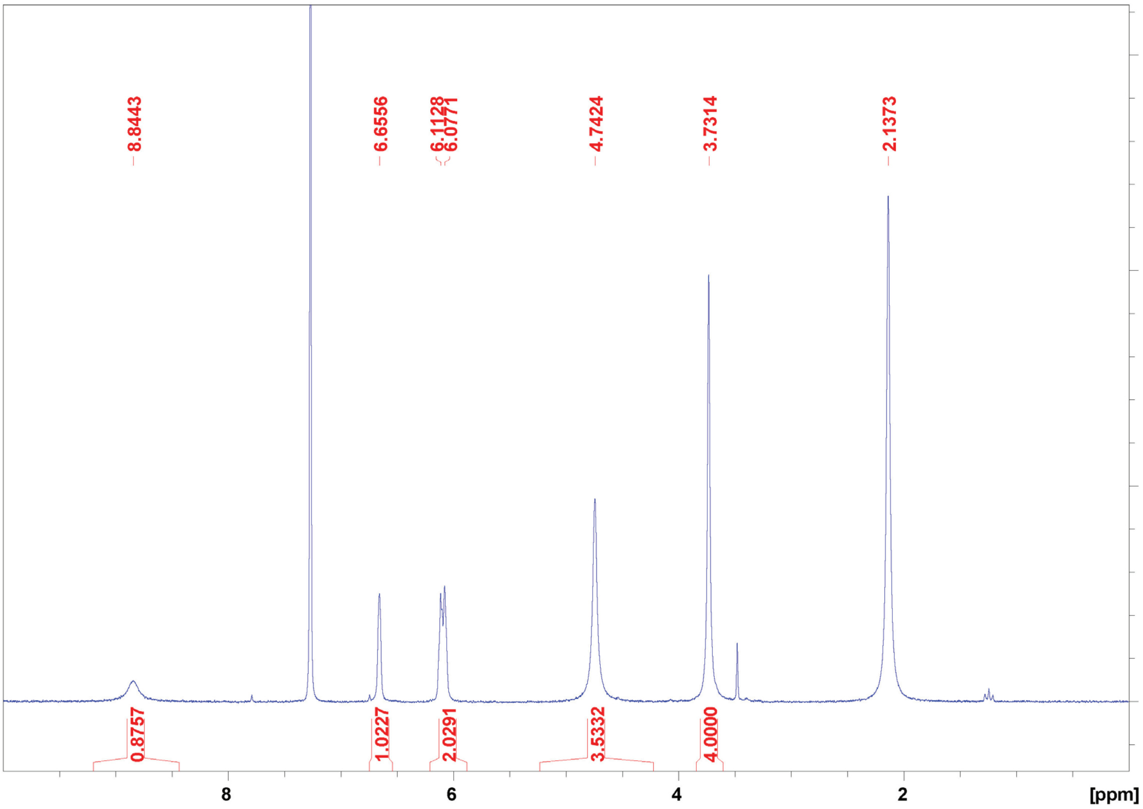

Figure S4. ^{1}H NMR spectrum (200 MHz, $CDCl_3$) of **IDS060**.

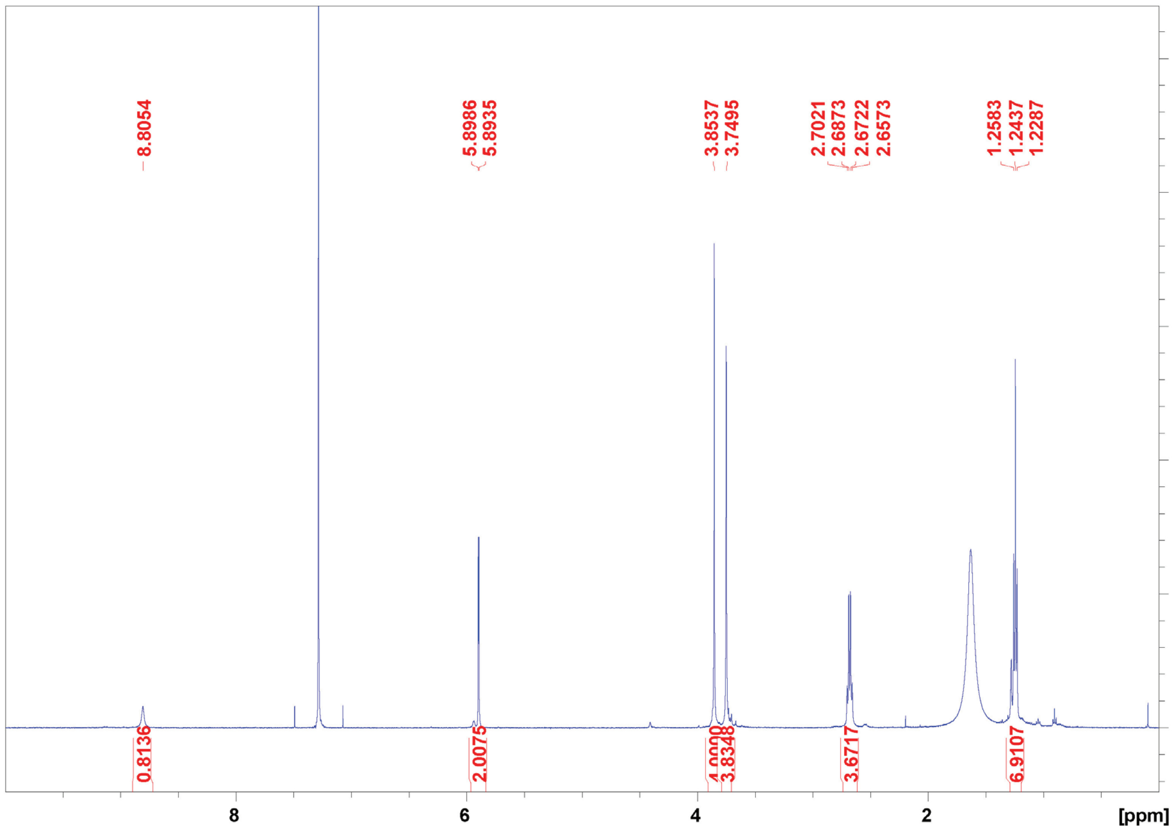

Figure S5. ^{1}H NMR spectrum (500 MHz, $CDCl_3$) of **MAF075**.

Assigned modifications	Total glycan composition	Confidence level
1021.3598 (T323)	$Hex_2HexNAc_2NeuAc_1$	1
1062.3864	$Hex_1HexNAc_3NeuAc_1$	3
1095.3966	$Hex_3HexNAc_3$	3
1167.4177 (T323)	$Hex_2HexNAc_2NeuAc_1Fuc_1$	1
1312.4552 (T323)	$Hex_2HexNAc_2NeuAc_2$	1
1370.4971	$Hex_2HexNAc_3NeuAc_1Fuc_1$	3
1386.4920	$Hex_3HexNAc_3NeuAc_1$	3
1458.5131 (T323)	$Hex_2HexNAc_2NeuAc_2Fuc_1$	1
1515.5346	$Hex_2HexNAc_3NeuAc_2$	3
1532.5499	$Hex_3HexNAc_3NeuAc_1Fuc_1$	3
1589.5714	$Hex_3HexNAc_4NeuAc_1$	3
1823.6453	$Hex_3HexNAc_3NeuAc_2Fuc_1$	3
1968.6828	$Hex_3HexNAc_3NeuAc_3$	3
203.0794 (T323)	$HexNAc_1$	1
203.0794 (T323)<br>203.0794 (S325)	$HexNAc_2$	2
203.0794 (T323)	$Hex_1HexNAc_3NeuAc_1$	1

859.3070 (S325)		
365.1322 (T323)	$Hex_1HexNAc_1$	1
365.1322 (T323)<br>1167.4177 (S325)	$Hex_3HexNAc_3NeuAc_1Fuc_1$	1
365.1322 (T323)<br>859.3070 (S325)	$Hex_2HexNAc_3NeuAc_1$	1
494.1748 (T323)	$HexNAc_1NeuAc_1$	1
494.1748 (T323)<br>494.1748 (S325)	$HexNAc_2NeuAc_2$	2
568.2116 (T323)	$Hex_1HexNAc_2$	1
656.2276 (T323)	$Hex_1HexNAc_1NeuAc_1$	1
656.2276 (T323)<br>1021.3598 (S325)	$Hex_3HexNAc_3NeuAc_2$	1
656.2276 (T323)<br>1167.4177 (S325)	$Hex_3HexNAc_3NeuAc_2Fuc_1$	1
656.2276 (T323)<br>568.2116 (S325)	$Hex_2HexNAc_3NeuAc_1$	1
656.2276 (T323)<br>730.2644 (S325)	$Hex_3HexNAc_3NeuAc_1$	1
730.2644 (T323)	$Hex_2HexNAc_2$	1
730.2644 (T323)<br>494.1748 (S325)	$Hex_2HexNAc_3NeuAc_1$	2
771.2909	$Hex_1HexNAc_3$	3
859.3070 (T323)	$Hex_1HexNAc_2NeuAc_1$	1
859.3070 (T323)<br>365.1322 (S325)	$Hex_2HexNAc_3NeuAc_1$	1
933.3438	$Hex_2HexNAc_3$	3
947.3230 (T323)	$Hex_1HexNAc_1NeuAc_2$	1

Table S1: Assigned O-glycosylations on peptide 320-328 (VQPTESIVR). The assignments include the observed mass shift for each modification, and the identified glycosylation sites in brackets. The localization is included only when identified with confidence level 1 or 2. A putative glycan composition is reported, based on the exact mass shift of the modification.
